# Epidemiology of Cerebro-Spinal Fever

**Published:** 1919

**Authors:** 


**Epidemiology of Cerebro-Spinal Fever.** By Lieut. J. T. Short. *U. S. Naval Medical Bulletin*, October 1918.

Whenever a case of cerebro-spinal fever was diagnosed, the company from which the patient came was quarantined and every one cultured; additional cultures were made of all possible contacts, such as hospital corps men, etc. Upon completion of a preliminary examination of the cultures, usually 24 hours, the men who had yielded organisms resembling the meningococcus in cultural characteristics, morphology, and staining reactions, were declared to be carrier suspects and placed in isolation units or " cubicles ". Quarantine was lifted and remaining men discharged to duty. A final laboratory report based on agglutination reactions was made, and those whose cultures gave positive reactions were sent to the meningococcus-carrier camp. No attempt was made to distinguish types of meningococci. Flexner's polyvalent serum was used for agglutination tests.

Isolated carriers were cultured at intervals of five days, and held until four successive negative cultures were obtained.

In taking cultures the open-swab method was used, the medium being peptone blood-agar.

In December 1917, 19 cases of cerebro-spinal fever were reported at the Naval Training Station; epidemic proper began in January with 84 cases; incidence decreased during the last week; and February concluded the epidemic with 10 in the first three weeks. Mortality was about 22 per cent. with a marked decrease after the middle of January. Almost exactly coincident with the epidemic in the latter part of December and January, there was a long-continued period of cold weather. Notes from various army camps during the winter show that the cold weather also influenced the meningitis incidence.

The great majority of men affected were young, unseasoned recruits who had been rapidly rushed through the regular incoming detention camp at a time when the station was rapidly expanding, and sent to already crowded camps where they were given hard work, and were exposed to severe cold and stormy weather. While with one exception the unseasoned recruits did not develop the disease in the regular detention camp where they were more carefully cared for, they were unable to withstand the combination of circumstances and depressing influences met in the other camps, although these same factors, operating on all alike, did not materially influence the older men at the station.

In regard to carriers and their relation to the epidemic, the author concludes that : —

The nearly ideal application of a carrier segregation system failed to prevent or appreciably affect an epidemic which ran its course and subsided with the cold weather after general hygienic measures had been instituted.

All of the real carriers present are never discovered at any one time, even with the most careful technic. This is best shown in the routine culture of chronic carriers where it is seldom possible to get more than 50 per cent. of cultures positive.

Carriers cannot be traced in relation to new cases, except in a very few instances, and they are not always closely associated with the old ones. New cases occur or fail to occur without apparent reference to carriers who remain or who are taken away.

It has been found in general by most observers that the segregation and treatment of carriers does not give satisfactory results in hastening the disappearence of the organism.

During the past nine months, 1,363 carriers have been found at this station, and 120 cases of cerebro-spinal fever have developed. During one month only has the number of cases exceeded 20, but from 200 to 400 men have been in isolation constantly. When the efficiency of the general measure is not clearly evident, it does not seem consistent with military economy to deprive the organization of 10 men continuously for every one who it is hoped will be protected from a temporary disability.

The search for meningococcus carriers and their disposition should not cause neglect of the more generally effective and easily controlled measures of general hygiene.

Haste, crowding, exposure and exhaustion in training recruits should be avoided. Other sanitary measures recommended are : —

The men who have been in indoor contact with a cerebro-spinal fever patient should be quarantined in as small groups as possible in commodious barracks, with good ventilation and ready access to available sunshine. Naso-pharyngeal sprays with an antiseptic solution should be given at regular intervals. Special attention and treatment should be given men with " colds " and coughs, including instruction in properly screening others from an unavoidable shower of saliva. Curtains or screens may be placed between the hammocks or beds at nights. Light exercises may be taken outdoors with proper clothing and protection from the weather. After five days or a week, when the temporary carriers will probably have cleared up and the chronic carriers become relatively harmless, the whole group may be discharged to duty. During an epidemic, large and promiscuous gatherings should be prohibited.

During a quarantine, cultures may be taken and repeated, with a preceding temporary suspension of antiseptics, and, as a middle course during an epidemic, chronic carriers might be segregated to the extent of preventing contact with others indoors.

All usual precautions should be taken in isolating and handling the sick; attendants should wear masks. Recognition of mild and previously unrecognized cases and their prompt treatment will do much to prevent outbreaks.

